# Biological and Mechanical Properties of Denture Base Material as a Vehicle for Novel Hydroxyapatite Nanoparticles Loaded with Drug

**DOI:** 10.34172/apb.2021.009

**Published:** 2020-11-07

**Authors:** Asmaa Nabil Elboraey, Hanan Hassan Abo-Almaged, Ahmed Abd El-Rahman El-Ashmawy, Aya Rashad Abdou, Amani Ramadan Moussa, Laila Hassanian Emara, Hossam Mohammed El-Masry, Gehan El-Tabie El Bassyouni, Magda Ismail Ramzy

**Affiliations:** ^1^Fixed and Removable Prosthodontics Department, National Research Centre, 33 El Buhouth Street, Dokki, P.O.12622 Cairo, Egypt.; ^2^Refractories, Ceramics and Building Materials Department, National Research Centre, 33 El Buhouth Street, Dokki, P.O.12622, Cairo, Egypt.; ^3^Medicinal and Pharmaceutical Chemistry Department, Pharmaceutical and Drug Industries Research Division, National Research Centre, 33 EL Bohouth st. (former EL Tahrir st.), Dokki, Giza, Egypt, P.O.12622, Affiliation ID: 10014618.; ^4^Chemistry of Natural Microbial Products Department, National Research Centre,33 El Buhouth Street, Dokki, P.O.12622, Cairo, Egypt.

**Keywords:** PMMA discs, Nanocarriers, HP-NP, Drug delivery, Cytotoxicity, Surface micro-hardness

## Abstract

***Purpose:*** This study aimed to evaluate the biological and mechanical properties of the poly(methyl methacrylate) (PMMA) denture base material as a vehicle incorporating novel hydroxyapatite nanoparticles (HA-NP) loaded with metronidazole (MZ) drug.

***Methods:*** HA-NP was prepared via wet-chemical-method, characterized by XRD, SEM/EDX, TEM, Fourier-transform infrared spectroscopy (FTIR), as well as the measurement of surface area and pore-size distribution. Four drug delivery formulas were prepared in the form of discs (10 x 2 mm) as follows: F1 (MZ/ HA-NP/PMMA), F2 (HA-NP/ PMMA), F3 (control-PMMA) and F4 (MZ/PMMA). Characterization of all formulas was performed using differential scanning calorimetry (DSC) and FTIR. MZ release rate, antimicrobial properties against three oral pathogens, cytotoxicity (MTT assay) and surface micro-hardness were also assessed. Statistical analysis of data was performed using one-way ANOVA test (*P* < 0.05).

***Results:*** DSC thermograms showed compatibility among MZ, HA-NP and PMMA along with physical stability over 6 months storage period at room temperature. FTIR spectroscopy proved the absence of any possible chemical interaction with MZ. MZ-HA-NP/PMMA formula showed relatively better drug release compared to MZ-PMMA. Both formulas showed statistically significant antimicrobial potentials against two microbial strains. MTT demonstrated reduction in cell cytotoxicity after 96 hours with the least value for HA-NP. Surface micro-hardness revealed non-significant reduction compared with the control PMMA.

***Conclusion:*** A novel biocompatible drug nanocarrier (HA-NP) was developed and incorporated in PMMA denture base material as a vehicle to allow prolonged sustained drug release to manage oral infections.

## Introduction

The need for removable prostheses will continue due to the increase in human aged population.^1^ Poly(methyl methacrylate) (PMMA) is the denture based material of choice for more than 50 years and up to now owing to its applicable mechanical, physiochemical, and working properties.^2,3^ However, denture PMMA may be colonized by some microorganisms such as*Candida albicans*, *Streptococcus mutans* and *Staphylococcus aureus*. Such oral pathogens may contribute to oral infections as denture stomatitis, systemic infections and pneumonia.^4^ Denture cleansers alone are ineffective against denture plaque removal and also, may cause significant damage to dentures.^5^

Systemic antibiotic has been specified for management of oral infections; however, excessive use of systemic drugs led to the development of microbial resistant strains.^6^ On the other hand, the topical application of antibiotics may be ineffective due to its inability to maintain physical contact with oral mucosal tissue, limiting anatomical features of the oral cavity as well as constant washing effect caused by the salivary flow.^7^ Therefore, there is a need for a technology that permits controlled drug delivery with optimal concentration at the required site.^8^

Recently, the introduction of nanoparticle-based drug delivery technologies allows an effective and targeted drug delivery without undesirable side effects. The therapeutic applications of drug delivery include cancer therapy, antimicrobial actions and vaccine delivery. Such technology is more appropriate to patients as it does not require frequent application regimes. In addition, direct delivery of the drug to the site of infection reduces the risk of systemic side effects or drug-drug interactions.^9,10^ For a material to be used as a drug carrier, it must have the ability to carry a drug either physically or chemically. Drug carrier must preserve the drug until it reaches the targeted site, being progressively degraded, and deliver the drug in a controllable manner over time. Generally, drug nano-carriers were classified into non-biodegradable and biodegradable. Nanocarriers biodegradable category includes collagen, sponges, or inorganic nanoparticles as calcium phosphate.^11,12^ Hydroxyapatite exists naturally in the body, as a major constituent of bone and tooth enamel, which accounts for their superior biocompatibility and biodegradability.^13,14^

In literature, incorporation of different antimicrobial agent into denture base resins such as: chlorhexidine, local antibiotics and nanomaterials as nano-silver, nano-titanium dioxide, or nano-silicon dioxide have been extensively studied.^15,16^ Meanwhile, the use of PMMA as vehicle for drug nanocarriers to ensure sustained release of drug and to control oral infection in removable prostheses was investigated by Mohamed Hamouda.^17^

Therefore, the aim of the present study was to develop and formulate biodegradable hydroxyapatite nanoparticles (HA-NP) as a nanocarrier for metronidazole (MZ) as a model drug using PMMA resin as a vehicle to manage the oral infection.

## Materials and Methods

### Materials

1. Diammonium hydrogen phosphate, Merck, Germany
2. Calcium nitrate tetrahydrate (extra pure), Acros, New Jersey, USA
3. Ammonia hydroxide, LOBA Chemie, Mumbai, India
4. Chemically activated PMMA (Acrostone, dental and medical suppliers, industrial zone, Cairo -Egypt)
5. Metronidazole, (Batch No: 0181901176, HUANGGANG, HUBEI PROVINCE, CHINE.)
6. HEPES Buffer (Lot No: S17706L0180, Serox GmbH, Mannheim, Germany)
7. Sodium Azide, (Batch No: L215501802, LOBA CHEMIE PVT.LTD Mumbai, India.)
8. Potassium bromide (Lot# BcBW6864, Sigma Aldrich, Germany)
9. Dulbecco’s Modified Eagle’s Medium Ham’s F-12 (DMEM/F12) cell culture medium (Lonza, Veries, Belgium).

### Preparation and characterization of HA-NP

HA-NP was prepared via wet chemical method by the reaction between calcium nitrate tetra hydrate and diammonium hydrogen phosphate.^11^ The stoichiometric ratio of calcium nitrate [Ca(NO_3_)_2_.4H_2_O] and diammonium hydrogen phosphate [(NH_4_)_2_HPO_4_] was adjusted to obtain a final (Ca/P) molar ratio at about 1.68. HA was precipitated as a milky gelatinous precipitate through the slow addition of the (0.6M) of (NH_4_)_2_HPO_4_ to (1M) of [Ca(NO_3_)_2_.4H_2_O] while stirring. To adjust the pH of the mixture at 11, ammonium hydroxide (NH_4_OH) solution was gradually added. The precipitate was left to be digested under reflux for 1hour at 65°C, washed to remove the NH_4_^+^ and NO_3_^–^ and then separated by centrifugation. The obtained powder was dried overnight at 110°C. The resulting amorphous product was calcined in an electrical furnace at 500°C for 2 hours at a heating rate of 10°C/min in air.

To identify the crystalline phases of the HA powder, X-rays diffraction analysis was applied using XRD [Bruker D8 Advance, Germany], Cu-Kα radiation (λ = 0.15418 nm), Ni filter. XRD diffractometer operated at 40 kV and 40 mA over a 2θ range of 10°-70°. Likewise, the crystallinity and nanoparticle nature of the HA powder were examined by high resolution transmission electron microscope (HR-TEM) model (JEOL JEM-2100, Japan). TEM samples were prepared by dispersing HA-NP in distilled water then, processed in an ultrasonic transducer for 30 minutes. A single drop of the prepared suspension was drop-casted onto a carbon-coated copper grid, air dried at room temperature for 40 minutes, before being microscopically analyzed.^18^

To study the crystal morphology, microstructure details and elemental analysis of the prepared nano powder, scanning electron microscopy (SEM) model FEI, QUANTA, FEG, 250 equipped with energy dispersive X-ray analysis unit (EDX) was used. Samples were sputter coated with a thin film of gold to ensure a high quality image. Fourier transform infrared spectroscopy (FT/IR- 6100) was used to examine the chemical composition and define the functional groups using the KBr pellet technique over the range of 400-4000 cm^-1^. The Barrett-Emmett-Teller method was employed to determine the surface area of the prepared HA powder. It was carried out by Nitrogen adsorption-desorption isotherm (Bel Sorb Max device from BEL, Japan INC). Total pore volume was calculated using the single point adsorption method at relative pressure of (p/p_0_ = 0.932). Furthermore, the pore size distribution was estimated using mercury porosimeter (Pore Size, Micromeritics model 9320, USA).

### Preparation and characterization of local delivery formulas

#### Preparation of PMMA local delivery disc specimens

Chemically activated PMMA (Acrostone, dental and medical suppliers, industrial zone, Cairo, Egypt) supplied in the form of polymer powder and monomer liquid was selected for this study. PMMA was used as vehicle for both MZ and nanocarrier loaded with MZ. For control PMMA (F3), the disc of dimension (10 mm diameter X 2 mm thickness) were manually prepared by mixing powder to monomer according to manufacture (2.8 mg polymer powder/ 1.2 mL liquid monomer).^19^ When dough stage was reached, the mixture was packed into a specially fabricated hexagonal stainless-steel molds, with inner detachable Teflon rings and sandwiched between two glass slabs then allowed to polymerize for 15 minutes. Other three PMMA drug delivery discs were prepared by incorporating different weight percentages of MZ, HA-NP and combination of MZ and HA-NP to the PMMA polymer powder as shown in Table 1.

**Table 1:** PMMA Local Drug Delivery Discs

**Formula Code**	**Disc Composition (% w/w)**		
	**MZ**	**HA-NP**	**PMMA**
F1	10	10	80
F2	0	10	90
F3 (control)	0	0	100
F4	10	0	90

Then the monomer was added to PMMA powder, thoroughly mixed following previous procedure to obtain the three drug formulas.

### Characterization of PMMA local delivery discs

Differential scanning calorimetry (DSC): DSC was performed for pure MZ, PMMA, HA-NP and crushed discs of each batch to detect possible chemical interactions between the drug and the excipients employed in disc formulations. DSC 131 evo (SETARAM Inc., France) was used to implement the differential scanning calorimeter analysis. DSC instrument was calibrated using the standards (Mercury, Indium, Tin, Lead, Zinc and Aluminum). Nitrogen and helium were used as purging gases. Test was programmed starting from 25-250°C. 120 μL of the samples were weighted in an aluminum crucible and introduced into the DSC. The sample was heated up to 250°C at a heating rate of 10°C/min. Thermogram results were processed using (CALISTO Data processing software v.149).

Fourier transform-infrared (FTIR) Analysis:FTIR spectra were obtained using FT-IR-6100 spectrometer (Jasco, Japan). The samples (pure MZ, PMMA, HA-NP and crushed discs of each batch) were ground and mixed thoroughly with potassium bromide (KBr), an infrared transparent matrix, at the ratio of 1:5 (sample/KBr), respectively. The KBr disks were prepared by compressing the powders at a pressure of 5 tons for 5 min in a hydraulic press. Scans were obtained at a resolution of 4 cm^-1^, from 4000 cm^-1^ to 400 cm^-1^.

Stability study:Prepared discs were stored on bench under ambient conditions, in a tightly closed containers away from direct sunlight. After 6-months, the stored discs were analyzed using DSC and Fourier-transform infrared spectroscopy (FTIR)and compared to fresh samples.

### Assessment of release of metronidazole from different local delivery discs

Discs containing MZ drug (F1 and F4) were individually immersed in 5 mL buffer (artificial saliva) containing physiological concentrations of electrolytes in saliva (0.7 mole/L CaCl_2_, 2.6 mole/L MgCl_2_ 0.2 mole/L H_2_O, 4.0 mole/L KH_2_PO_4_, 30 mole/L KCl, 0.3 mole/L NaN_3_, 20 mole/L of HEPES buffer) with slight viscosity at neutral pH.^20,21^ The specimens were shoken at 37°C and 40 RPM using Temperature-controlled shaking water-bath, Lab-Line,Dubuque, IA, USA. The artificial saliva was daily replaced. Periodically, the quantity of drug released from each specimen was determined by UV spectrophotometry (UV-Visible spectrophotometer, Beckman, DU-650, USA) measurements at λ_max_ = 321 nm. Each test was repeated 5 times and MZ release profile was tested for a one month period.^22^

### Antimicrobial assessment of the PMMA local delivery discs

The antimicrobial properties of the two prepared discs containing MZ (F1 and F4) were assessed using shaking flask method. Discs were soaked in 5.0 mL artificial saliva and incubated in a shaker incubator at 37°C for different periods of 2, 4 and 8 days. Three pathogenic Gram-positive bacterial strains were tested including: *Staphylococcus aureus* (ATCC 6538), *Enterococcus faecalis* (ATCC 19433) and *Streptococcus mutans* (ATCC 25175). The antibacterial test was performed quantitatively using standard test method according to the AATCC test method 100-1999 for Bacterial Counting.^23^ Each bacterial strain was individually inoculated into 5 ml BHI (brain heart infusion broth) sterile suspension. The suspension was adjusted to 0.5 McFarland standards to match the turbidity of 1.5 × 10^-8^ mL^-1^.^24^ In a shake flask, 1 mL of elute from each formula was added to each bacterial strain and incubated at 37°C for 24 hours. All experiments were performed in triplicates to have adequate statistical power to make the observations from that study expressive. The antibacterial activity was expressed in reduction (%) of the organisms after getting in contact with the test specimen compared with the number of the organism cells surviving after contact with the control (F3).^23^ The percentage of pathogenic microorganism reduction after contact with the tested formulas was measured using spectrophotometer (Perkin-Elmer Lambda EZ 201 UV-Vis-USA) and compared with control bacterial strain. All results were expressed according to the following equation:

(1) Reduction(%)=A−BA100

A: The number of microorganisms present on control flask contains bacterial strain only without any thing.

B: The number of microorganisms present on shake flask after applying tested micro-organism samples.

### Cytotoxic assessment of different PMMA local delivery discs

The potential cytotoxicity of different drug delivery discs (F1, F2, and F4) as well as the control one (F3) were determined using 3-(4,5-dimethylthiazol-2-yl)-2,5-diphenyl tetrazolium bromide (MTT) assay test. The test was conducted on human normal fibroblast cell (BJ1) in Bioassay-Cell Culture Laboratory (National Research Centre, Cairo, Egypt). All test steps were performed in a laminar flow cabinet biosafety class II level (Baker, SG403INT, Stanford, ME, USA). Each formula was immersed in 100 mL of Dulbecco’s Modified Eagle’s Medium Ham’s F-12 (DMEM/F12) cell culture medium (1:1 MIX Lonza, Veries, Belgium) for 24, 48 and 96 hours. Cytotoxicity of the specimen elutes were assessed, after each immersion period. The BJ1 cells were suspended in DMEM/F12, 1% antibiotic-antimycotic mixture (10 000 U/mL potassium penicillin, 10 000 µg/mL Streptomycin Sulfate and 25 µg/mL Amphotericin B) and 1% L-glutamine at 37ºC under 5% CO_2_. Cells were batch cultured for 10 days and seeded at concentration of 10 x 10^3^ cells/well in a new growth medium into 96-well microtiter plastic plates at 37ºC for 24 hours under 5% CO_2_ using water jacketed carbon dioxide incubator (Sheldon, TC2323, Cornelius, OR, USA).

After 24 hours the media was replaced with equal volumes of elute materials from both composite specimens’ and tissue conditioning. For negative control, cells were incubated in DMEM/F12 alone. Although, positive control 100 µg/mL of a natural agent of known cytotoxic effects (100% lethality) under the same conditions was used.^25,26^

After 48 hours of incubation, the medium was replaced with 40 ul MTT salt (2.5 μg/mL) and incubated for further 4 hours at 37ºC under 5% CO_2._ To stop the reaction and dissolve the formed crystals, 200 μL of the 10% sodium dodecyl sulphate in deionized water was added then incubated overnight at 37°C. Absorbance was measured using a microplate multi-well reader (Bio-Rad Laboratories Inc., model 3350, Hercules, California, USA) at 595 nm and a reference wavelength of 620 nm. The percentage of change in viability was calculated according to the formula:

(2) Reading of specimensReading of negative control−1100

### Surface micro-hardness of PMMA local delivery discs

36 specimens of F1, F2, control F3 and F4 (n = 9) were prepared to measure the surface micro-hardness using Vicker’s hardness testing machine (Nexus 4503, INNOVATEST, Netherlands, Europe). A 100 g load was applied for 30 seconds with 20X magnification. Every disc was subjected to three indentations (one on the center and two on the borders) and the average value was recorded.^19^

### Statistical analysis

Data were analyzed using IBM^®^ SPSS^®^ (SPSS Inc., IBM Corporation, USA) statistics version 23 for Windows. One-way repeated measures ANOVA test followed by Tukey’s post hoc was used for comparison between different formulas and during the follow up periods within each formula. The significant difference was set at (*P* < 0.05)

## Results and Discussion

### Characterization of HA-NP

**Figure 1:**
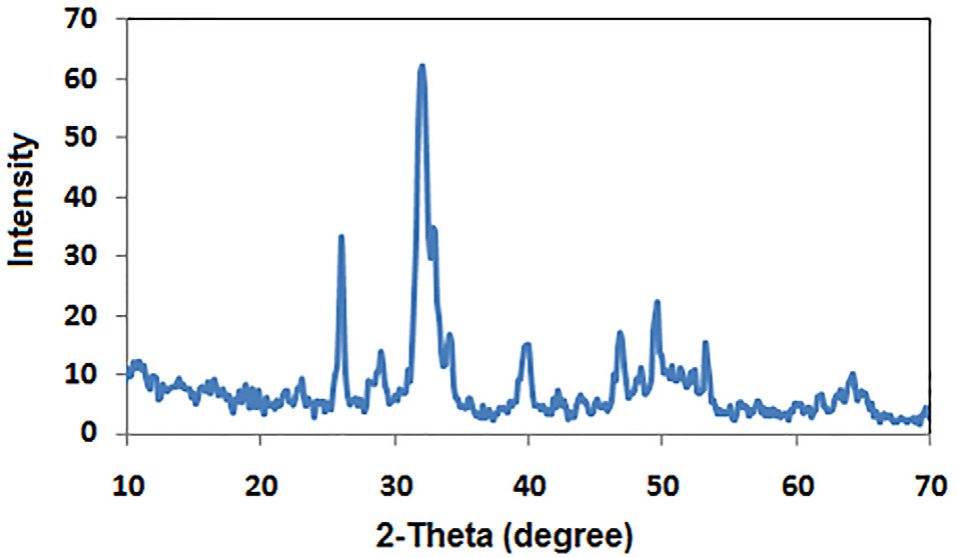
XRD pattern of the prepared HA-NP powder.

**Figure 2:**
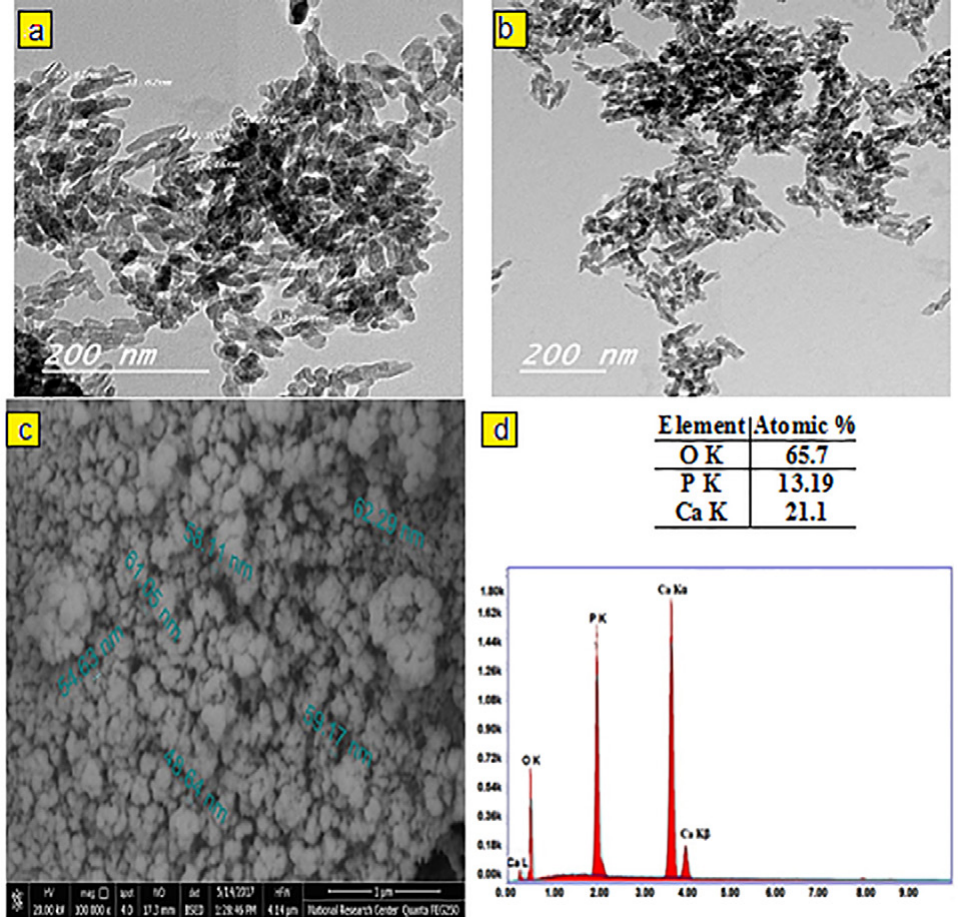
The TEM micrographs (a, b), the SEM micrographs (c) and (d) EDX analysis of the prepared HA-NP powder.

**Figure 3:**
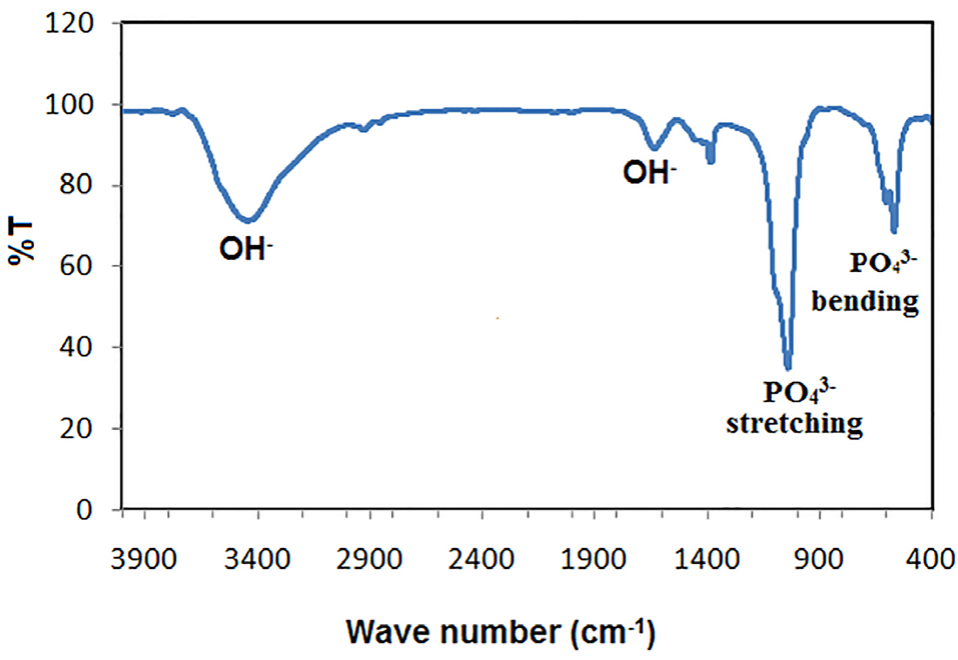
FTIR spectrum of the synthesized HA-NP powder.

The crystal phase composition of the prepared hydroxyapatite powder was determined using the X-ray diffraction (XRD) pattern (Figure 1). Peak matching was done by comparing respective 2θ values to standard peak values in the International Centre for Diffraction Data (ICDD) cards of HA standard pattern. Results revealed the presence of all major HA peaks at 2θ ∼31.8°, 2θ ∼32.7° and 34.2° in a good agreement with the ICDD standard pattern for the hydroxyapatite (JCPDS card no. 76-0694). The absence of peaks at 2θ =17-18° and 19-21° proves the lack of other secondary phases such as CaO and α-TCP, respectively.^12^ TEM micrographs of the synthesized HA-NP powder were shown in (Figure 2a & b). The average particle size of the HA grains was in the nano range of 11-36 nm (Figure 2a). The grains appeared in a nano-rod morphology; with uniform size aggregated together in the form of clusters, (Figure 2b). Ikeda et alproposed that the rod-shaped HA crystals in bone substitute applications are more favored, owing to the effective osteoclast homing activity and local bone metabolism of the rod-shaped HA crystals.^27^ EDX analysis for the HA-NP powder was shown in (Figure 2d). Ca/P ratio was around 1.59. SEM micrograph displayed in (Figure 2c), showed a uniform and homogeneous microstructure network with very fine agglomerate particles in the nanometer size range between 30-56 nm. Particles displayed porous and interconnected morphology consequently, signifying it can be used for bone tissue engineering applications.^28^ The functional groups of the HA-NP powder were identified using the FTIR spectrum (Figure 3). Bands at 3433 cm^-1^ and 1632 cm^-1^, related to the OH- stretching and bending vibrations, respectively. The strong band at 1039 cm^-1^ was attributed to the symmetric stretching vibrations of υ_3_PO_4_- group. Characteristic bands indicative of bending vibration of υ_4_PO_4-_ appeared as a doublet at 604 and 569 cm^-1^.^29,30^ The carbonate bands detected at 1450 and 875 cm^−1^ assigned to the stretching and bending vibrations of the CO_3-_ ion owing to the incorporation of CO_3-_ groups for the PO_4_- sites in the structure.^31^ These groups may have been adsorbed from the atmosphere by the HA during the synthesis process.^32^ In this study, the FTIR spectra matched to those reported by previous authors and are consistent with the XRD results.^33,34^ The prepared HA-NP showed a surface area of 131.9 (m^2^/g). Total pore volume was 0.1014 cm^3^/g, whereas the total pore size distribution of the prepared HA-NP ranged between 9-14 nm with a mean pore diameter of 11.2 nm.

### Characterization of PMMA local delivery disc specimens

Chemically cured resin was selected to ease manipulation, since it requires no special equipment. As well as to standardize the laboratory procedures during the preparation of different drug formulas thus eliminating possible lab technician inaccuracies. Four drug delivery formulas were prepared F1 (MZ/HA-NP/PMMA), F2 (HA-NP/PMMA), F4 (MZ/PMMA), in addition to F3 (control/PMMA). Only 10% weight percentage of MZ drug powder was added to PMMA since this weight percentage had no detrimental effect on the properties of the PMMA.^35,36^ Similarly, 10% weight of HA-NP powder was also incorporated into PMMA. For F1 drug formula the maximum weight% of MZ and HA-NP added to PMMA without affecting manipulation characteristics of the PMMA resin was found to be 20%.

#### DSC analysis

**Figure 4:**
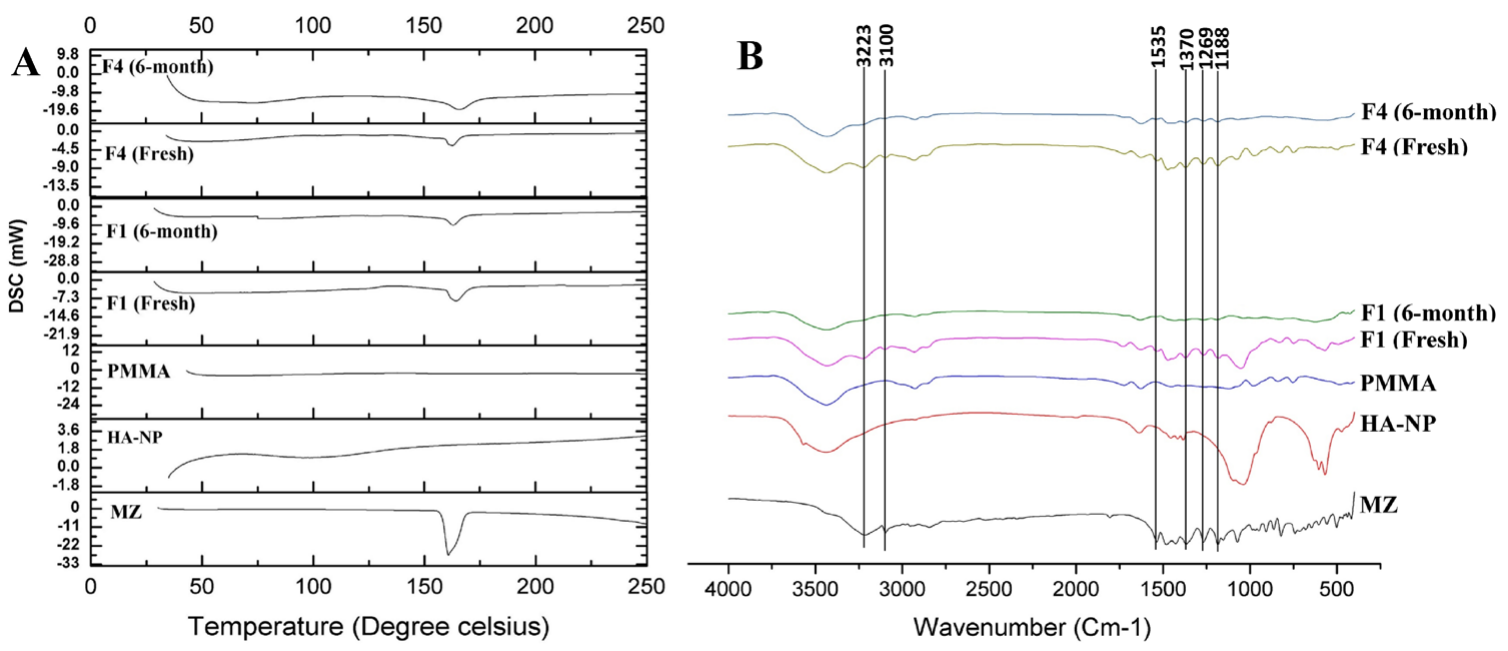
(a) DSC thermograms of MZ, PMMA, HA-NP, crushed prepared discs (fresh and 6-month stored samples). (b) FTIR spectra of MZ, PMMA, HA-NP, crushed discs (fresh and 6-month stored samples).

DSC measures the heat loss or gain resulting from physical or chemical changes within a sample as a function of temperature.^37^ Any possible drug - excipients interactions in the drug formulation could be predicted by conducting the DSC studies.^38^ Figure 4a showed the DSC thermograms of MZ, PMMA, HA-NP, crushed prepared discs (fresh and 6-month stored samples). A single sharp endothermic peak appeared at 166ºC represents the melting point of pure MZ.^39^ The appearance of the characteristic MZ melting endothermic peak in the prepared discs indicated absence of any possible interaction from PMMA (Figure 4a). The peak intensity of MZ in the discs showed a decrease in comparison with the sole drug, regarding the dilution effect of the formulation components.^40^ For drug sample and MZ-containing discs (F1 and F4 discs), the thermograms did not show any significant shift in endothermic peaks neither in the fresh nor in the stored discs. Based on the thermograms of DSC, there is no possibility of interactions between MZ and the proposed excipient. The prepared discs were physically stable over the 6 months storage period at room temperature.

#### FTIR analysis

The possibility of occurrence of any drug-excipients interactions in a drug formulation was also predicted by conducting the FTIR studies. Figure 4b showed the FTIR spectra of MZ, PMMA, HA-NP, crushed prepared discs (fresh and 6-months stored samples). Figure 4b showed the principle fingerprint of FTIR absorption bands for MZ at 3223, 3100, 1535 and 1370 cm^-1^ which recognizes the presence of C-H stretching, C=CH stretching, N-O stretching and NO_2_ symmetric stretching, respectively, in addition two strong characteristic bands at 1269 and 1188 cm^-1^ corresponding to O-H in the plan deformation and C-O stretching, respectively.^41^ The FTIR spectra of both the fresh and 6-month stored drug loaded discs (F1 and F4) exhibited all the characteristic bands as in the spectrum of the individual MZ excluding the possibility of any interaction (Figure 4b). Also, chemical and functional group changes during processing of the formulation of drug loaded discs were excluded.^42^ There was neither origin of any new characteristic peaks nor absence of any original characteristic peaks which revealed no incompatibility between drug and excipients even after storage.^43^ The appearance of the characteristic MZ bands indicated absence of any possible chemical interaction during formulation and storage.

### Drug release

**Figure 5:**
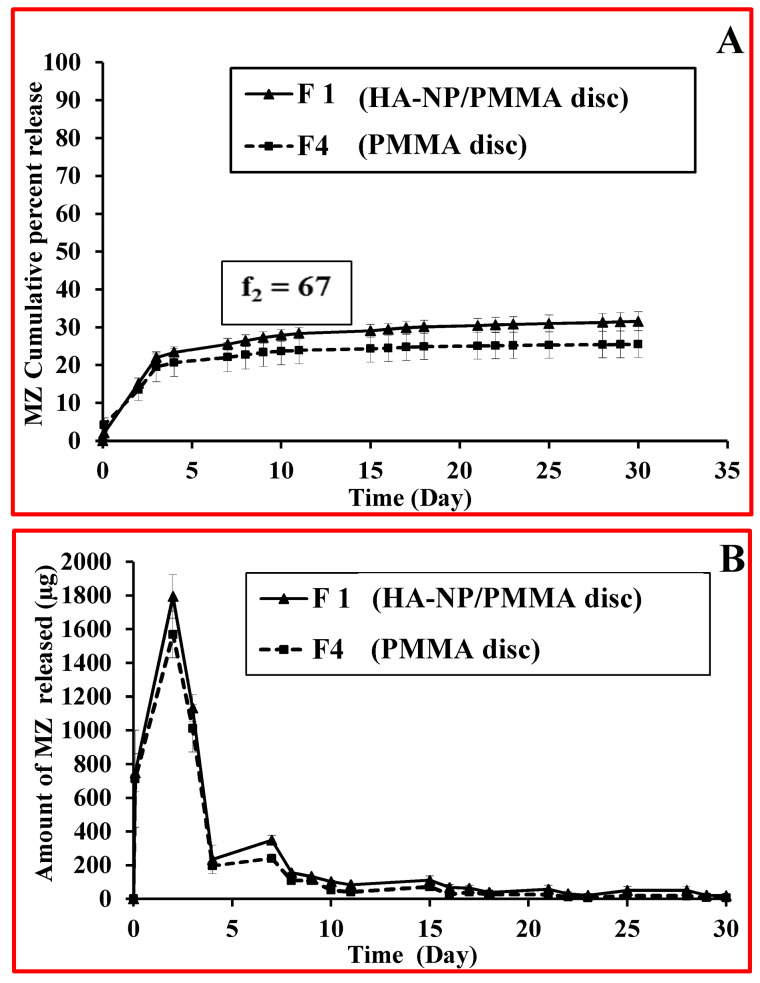
(a) MZ release profiles from PMMA discs in artificial saliva. (b) Amount of MZ released from PMMA discs in artificial saliva.

The antibacterial drug contained in the disc specimens, namely, MZ (20 mg/disc), demonstrated an initial high rate of release (burst effect) from the surface of PMMA discs during the first 3 days (22.06 % and 19.55 % for F1; HA-NP/PMMA discs and F4; PMMA discs, respectively) followed by a slower sustained release profile up to 30 days (31.59 % and 25.53 % for F1 and F4 discs, respectively) as shown in Figure 5a.^44-48^ MZ release profiles showed a biphasic release phenomenon. The first phase gave high amounts of drug released followed by a second phase of sustained release profile with a much lower release amount Figure 5b. The highest amount of MZ released was recorded on the second day (1794.62 µg and 1568.63 µg for F1 and F4 discs, respectively). Conferring to Dhana Lekshmi et al,^45^ the drug content which is closer to the surface of the particles was responsible for an increased initial burst and the drug in the core of particles is responsible for a prolonged drug release from the polymer.^47^ It is worthy to mention that the incorporation of HA-NP within the PMMA discs was able to relatively improve the MZ release from the resin discs, however resulting in a similar release profiles as depicted by the calculated “Similarity factor”, (ƒ_2_) value, whose value was 67. The dissolution profiles of F1 and F4 were compared by calculating the similarity factor (ƒ_2_) as proposed by Moore and Flanner^49^ which was defined using the following equation:

(3) f2=50log1−(1n)∑t=1nwt(Rt−Tt)2−0.5100

Where, R_t_ is the percentage of released drug for a reference batch at time point t, T_t_ is the percentage of released drug for the test batch, n: is the number of pull points collected during the in vitro release test, R_t_ and T_t_ are the cumulative percentages release at the selected time point of the two tested formulae. The Food and Drug Administration (FDA) set a public standard of (ƒ_2_) value of 50-100 to indicate similarity between two dissolution profiles (ƒ_2_).^50^ Future studies for tailoring the MZ resin discs in such a way to improve the release profile will be considered. Our strategies will be focusing on increasing polymer hydrophilicity by synthesizing functionalized PMMA microspheres or by formulating PMMA composites with higher ratios of hydrophilic polymers.

### Antimicrobial effect of the different local delivery discs

**Figure 6:**
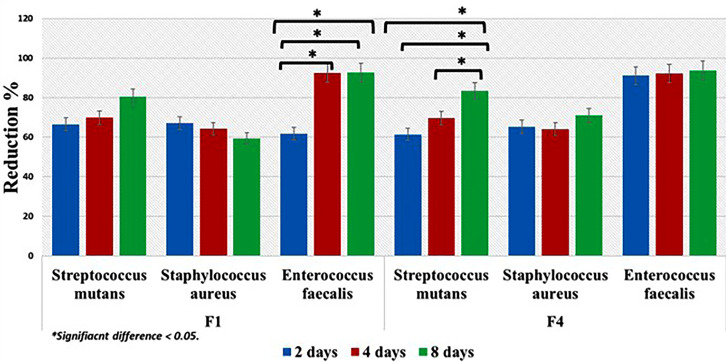
Percentage of bacterial reduction of the tested drug formulas.

Figure 6 showed the percentage of bacterial reduction of the tested drug formulas (F1 and F4). Although, the results demonstrated reduction in mean value of the tested strains over time reaching the maximum reduction percentages after 8 days. Comparison between F1 and F4 mean values showed no statistically significant difference (*P* > 0.05) except for *Enterococcus faecalis.* Wherever, F4 showed higher statistically significant mean value only after two days and the result was statistically significant (*P* < 0.05).

Regarding the antimicrobial activity of each drug formula, F1 demonstrated reduction in mean values after 2, 4 and 8 days. However, the results were not statistically significant (*P* > 0.05) expect against *Enterococcus faecalis* (*P* < 0.05). Although, F4 showed reduction in the mean value percentages, the results were statistically insignificant (*P* > 0.05) expect for *Streptococcus mutans* (*P* < 0.05)

Results demonstrated sustained gradual release of MZ drug from the two formulas (F1 and F4). Such outcome demonstrated that polymerization of the PMMA resin did not adversely affect the release of drug nor altering the diffusion characteristics of the resin. This finding was in agreement with previous studies that utilized polymers for delivering antimicrobial agent.^51,52^

The increase in the percentage of bacterial reduction when the elution of different drug formulas was added to each bacterial strain with time indicated that leaching behavior of MZ from each drug formal into artificial saliva was governed by a concentration dependent diffusion process.^35^

### Cytotoxic effect of the different local delivery discs

**Figure 7:**
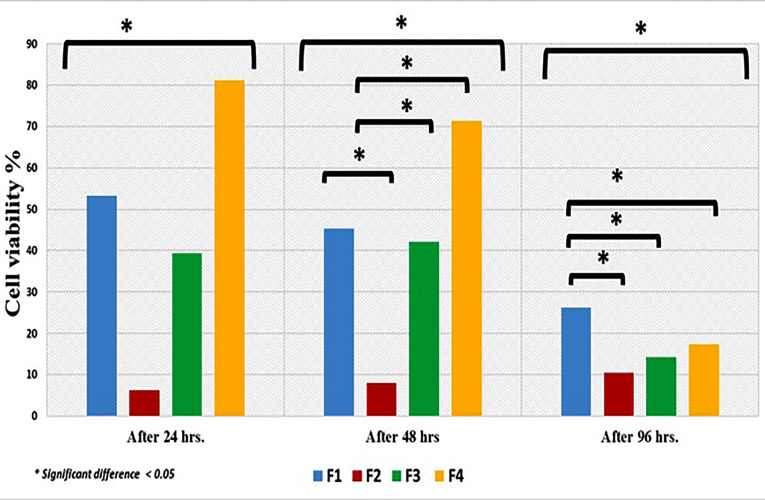
Cell cytotoxicity of the different drug formula.

The cytotoxic effect of the different formulas and control were shown in Figure 7. Comparison of the cell cytotoxicity mean values revealed that F1, F2, F4 and control F3 had cytotoxic effects and the results were statistically significant (*P* < 0.05) after 24 hours. Where F4 revealed highest cytotoxic mean values followed by F1, F3 and least mean value was for F2.

After 48 hours. There was reduction in cell cytotoxicity mean values and the results were statistically significant (*P* < 0.05). Again, the highest statistically significant mean value was recorded for F4 followed by F1, F3 and the least mean value was for F2 (*P* < 0.05).

Further reduction in cytotoxicity was evident after 96 hours. Wherever F1 showed highest mean value followed by F4, then F3 and finally F2 and results were statistically significant (*P* < 0.05).

The initial high cytotoxic effect of F1, F3 and F4 after 24 and 48 hours could be attributed to the leached unreacted PMMA monomer as documented in literature.^53,54^ For F4 this effect is coupled with the spurt release of MZ drug. On the other hand, F2 showed initially better biocompatibility compared to other formulas. The addition of 10% of HA-NP could possibly act as filler that primarily delayed the leaching of monomer in the first 24 and 48 hours. This was followed by a negligible increase in cytotoxicity.^50^ Such assumption also may explain the reduced cytotoxicity of F1 compared to F4 formula after 24 and 48 hours. Following 96 hours there was slight decrease in the mean values of all formulas and control indicating the cell recovery and reduction in cell cytotoxicity to the acceptable levels as reported by Chen et al.^55^

### Surface microhardness of different local delivery discs

Hardness is an important surface property used to predict the resistance of any dental material to penetrate and resist wear.^56^ The incorporation of MZ drug and nanocarriers to PMMA resin resulted in slight decrease in the surface hardness. Comparison of the surface microhardness of the control F3 (control PMMA) and three formulas F1, F2, and F4 were presented in Table 2. F3 showed higher mean value followed by F2 then F4 and the lowest value was for F1. However, there was no statistical significance difference between the control and tested formulas (*P* > 0.05).

**Table 2:** Surface microhardness of different drug disc

**Drug Delivery Formul**a	**M**	**SD**	***P*** **value**
F1	10.833^a^	3.5482	0.23*
F2	12.244^a^	3.0050	
F3 (control)	13.611^a^	1.2524	
F4	11.844^a^	2.8806	
Total	12.133^a^	2.8667	

M; mean, SD; sandard deviation. *Insignificant Difference. Values with same superscript letter in the same column were insignificant different.

This alteration in the hardness could be attributable to the decrease in polymer powder ratio in relation to the monomer content. The presence of excessive unreacted monomer adversely affects the mechanical properties due to its plasticizing effect that decreases the interchain forces and allowing the deformation to occur easily under load.^57^

## Conclusion

A novel drug delivery nanocarrier (HA-NP) was successfully developed that allowed sustained release of MZ drug over a prolonged period up to 1 month.

The developed nanocarrier was compatible physically and chemically with the MZ drug and PMMA denture base material.

Removable dental prosthesis fabricated from PMMA can be used as a vehicle for drug nanocarriers for management of oral infections. PMMA can incorporate additives up to 20% w/w (10% MZ and 10% HA-NP) without significantly altering the surface micro-hardness.

This study deserves to be extended with the same materials and additional excipients for better performance.

## Ethical Issues

This study was designed and approved by the Medical Research Ethical committee (MREC Approval No. 16085) of National Research Centre (NRC), Cairo-Egypt.

## Conflict of Interest

The authors declare that there are no conflicts of interest regarding the publication of this paper.

## Acknowledgments

We would like to thank Dr. Sherihan M. Eissa Researcher of removable prosthodontics for her efforts in preforming the statistics of this research. This research was financially supported by National Research Centre (NRC), Cairo, Egypt(Project grant no. P11010201).
